# Prevalence and Association of Polypharmacy and Potentially Inappropriate Medications Among Older Adults with Type 2 Diabetes Mellitus: A Systematic Review and Meta-Analysis

**DOI:** 10.3390/pharmacy14030065

**Published:** 2026-04-28

**Authors:** Raniah A. Aljaizani, Arwa A. Althumairi, Khalid A. Alamer, Shakil Ahmad

**Affiliations:** 1 Department of Pharmacy Practice, College of Pharmacy, Imam Abdulrahman Bin Faisal University, Dammam 31441, Saudi Arabia; kaalamer@iau.edu.sa; 2 Department of Health Information Management and Technology, College of Public Health, Imam Abdulrahman Bin Faisal University, Dammam 31441, Saudi Arabia; aalthumairi@iau.edu.sa; 3 Directorate of Library Affairs, Imam Abdulrahman Bin Faisal University, Dammam 31441, Saudi Arabia; shahmad@iau.edu.sa

**Keywords:** prevalence, polypharmacy, potentially inappropriate medication, older adults, type 2 diabetes mellitus

## Abstract

Older adults with type 2 diabetes mellitus (T2DM) are at high risk of polypharmacy and potentially inappropriate medication (PIM) use because of multimorbidity and complex treatment regimens. This systematic review and meta-analysis (PRISMA 2020; PROSPERO CRD420251149348) aimed to estimate the prevalence of polypharmacy and PIM use, describe PIM burden and patterns, and summarize the co-occurrence of PIMs among those with polypharmacy. On 7 September 2025, we searched PubMed, Scopus, Embase, Web of Science, and MEDLINE using a structured search strategy based on the PICO framework. Observational studies of older adults (≥65 years) with T2DM reporting polypharmacy and PIMs were included. Risk of bias was assessed using the JBI checklist, and prevalence estimates were synthesized using a random-effects meta-analysis. Five studies (13,350 participants) were included. Polypharmacy prevalence ranged from 43.6% to 95.3%, while PIM prevalence ranged from 23.4% to 74%. The co-occurrence of PIMs among polypharmacy users ranged from 39.6% to 74%. Commonly reported PIM classes included long-acting sulfonylureas, proton pump inhibitors, and benzodiazepines. Overall, polypharmacy and PIM use were frequently reported among older adults with T2DM; however, the wide variation in prevalence across studies indicates substantial clinical and methodological heterogeneity. These findings highlight the need for structured medication review and clinical context-based medication optimization beyond numerical thresholds.

## 1. Introduction

According to the International Diabetes Federation (IDF), 589 million adults aged 20–79 years have diabetes [1]. By 2050, this number is expected to increase to 853 million adults. Type 2 diabetes mellitus (T2DM) is the most prevalent form of diabetes, accounting for over 90% of all diabetes cases globally. T2DM is more common among older adults and often coexists with multiple comorbidities such as hypertension, dyslipidemia, cardiovascular diseases, and chronic kidney disease [2,3]. These cardiometabolic abnormalities cluster as metabolic syndrome, a recognized constellation of central obesity, hypertension, and dyslipidemia that contributes to multimorbidity and medication burden in T2DM [4,5,6]. Furthermore, age-related physiological changes alter pharmacokinetics and pharmacodynamics, including reduced renal and hepatic clearance, changes in body composition, and heightened drug sensitivity, thereby increasing susceptibility to adverse drug reactions [7]. These changes may precipitate prescribing cascades by further expanding medication regimens and complexity [8,9]. As regimens become more complex, older adults face a greater medication burden, increased risk of drug–drug interactions and adverse drug effects, adherence challenges, and safety risks [7,8,9,10]. Together, these factors make medication regimens challenging to optimize [11].

Polypharmacy is commonly defined as the concurrent use of five or more medications, though definitions vary widely and are often purely numerical rather than clinically contextualized [12]. Polypharmacy is increasingly recognized worldwide as a major challenge in chronic disease management, particularly among older adults [13,14]. Importantly, contemporary guidance distinguishes between inappropriate polypharmacy, in which potential harm outweighs benefits, and appropriate polypharmacy, in which multiple medications are prescribed and optimized according to best evidence for individuals with complex multimorbidity, aligned with patients’ clinical needs, goals of care, values, and preferences [15,16]. Among older adults with T2DM, polypharmacy is highly prevalent [17,18]. In this population, multimorbidity and age-related vulnerability can increase susceptibility to medication-related harms, including potentially inappropriate medication (PIM) exposure and other treatment-related risks, such as severe hypoglycemia, increased fall and fracture incidence, and hospitalization [5,17].

PIMs are medications where the risks outweigh the potential clinical benefits, especially when safer or more effective options are available, or when the medication is used without a clear indication [19,20,21,22]. Explicit screening criteria, most commonly the American Geriatrics Society (AGS) Beers Criteria and the Screening Tool of Older persons’ Prescriptions/Screening Tool to Alert to the Right Treatment (STOPP/START) criteria, are widely used to identify PIMs and support medication reviews [19,20,21,22]. Accordingly, among older adults with T2DM, there is a critical need to redefine polypharmacy, not only by focusing on medication count but also by considering the risk of PIM-associated polypharmacy [23].

Our search identified no synthesized evidence on the association between polypharmacy and PIM exposure within the same T2DM cohorts. Moreover, the burden and patterns of PIM use among individuals with polypharmacy have not been consistently synthesized. This highlights a vital research gap that limits clinicians’ and policymakers’ ability to benchmark risk and prioritize structured medication reviews. Studying polypharmacy and PIM together within the same cohorts provides added value by quantifying their association and identifying whether higher medication counts are linked to inappropriate prescribing insights that are not attainable when polypharmacy and PIM are examined separately.

Therefore, this systematic review and meta-analysis aimed to (1) estimate the pooled prevalence of polypharmacy among older adults with T2DM, (2) estimate the pooled prevalence of PIM use using validated explicit criteria (Beers and STOPP/START), (3) describe the burden and patterns of PIM exposure, and (4) narratively summarize the co-occurrence of PIMs among those with polypharmacy across the included studies.

## 2. Materials and Methods

### 2.1. Study Design and Reporting Guideline

This systematic review and meta-analysis synthesized the evidence on the prevalence of polypharmacy and PIM among older adults (≥65 years) with T2DM. The review was designed and followed the Preferred Reporting Items for Systematic Reviews and Meta-Analyses (PRISMA) 2020 statement [24]. The PRISMA checklist of this review is available in the Supplementary Material File S1.

### 2.2. Protocol Registration

Pre-specified outcomes include pooled prevalence estimates of polypharmacy and PIM use, and the association between polypharmacy and PIM exposure as defined prior in the registered protocol in the International Prospective Register of Systematic Reviews (PROSPERO September 2025; Registration No. CRD420251149348), which can be accessed online.

### 2.3. PICO Framework

The PICO framework was applied to define the eligibility criteria and to guide the development of the comprehensive database search strategy [25].

- Population (P): Older adults (≥65 years) with T2DM.
- Intervention (I): Polypharmacy (5 or more medications) and PIM based on validated explicit criteria, specifically the AGS Beers Criteria or the STOPP/START criteria.
- C: Non-polypharmacy.
- O: Prevalence and co-occurrence of polypharmacy and PIM among older adults with T2DM.

### 2.4. Eligibility Criteria

Inclusion criteria:

1. Studies reporting both the prevalence of polypharmacy and the prevalence of PIMs among older adults (≥65 years) with T2DM in any healthcare setting. We selected ≥65 years because it is the most used operational definition of “older adults” in geriatric and medication-safety research, including widely applied tools such as the Beers Criteria and the STOPP/START criteria, and is the most frequently reported age cut-off in the eligible literature. This enhances comparability across included studies and supports consistency in evidence synthesis [26,27].
2. Studies assessing the prevalence of PIMs using validated explicit criteria, specifically the AGS Beers Criteria or the STOPP/START criteria.
3. Studies including mixed populations (e.g., T2DM and other chronic conditions) will be included only if data for the T2DM subgroup are reported separately.
4. English language studies.

Exclusion criteria:

1. Studies not targeting older adults with T2DM.
2. Mixed population studies were excluded if T2DM-specific estimates could not be reliably extracted.
3. Studies assess polypharmacy or PIMs separately among older adults with T2DM.
4. Studies assessing PIMs using non-validated tools.
5. Studies focusing on type 1 diabetes, gestational diabetes, or prediabetes.
6. Studies focusing on pediatric or adolescent patients.
7. Studies where polypharmacy or PIMs are not clearly defined or measured.
8. Reviews, editorials, conference, book, case report, and case series.
9. Non-English-language studies.

### 2.5. Information Sources and Search Strategies

Five well-established medical databases were searched for relevant studies with no date restrictions: PubMed, Scopus, Embase, Web of Science, and Medline. A librarian specialized in systematic reviews guided the searching process. General search keywords were “older adults”, “type 2 diabetes”, “polypharmacy”, “potentially inappropriate medication”, “Beers”, “STOPP/START”, and “prevalence”, either in the title or abstract. For each database, a specialized search syntax was developed using free-text keywords in line with its strategies. In addition, automatic citation using Inciteful and CitationChaser was conducted to identify additional studies. The last search was conducted on 7 September 2025. The search syntax for this review is available in the Supplementary Material File S2.

### 2.6. Studies Screening Selection Process

All identified studies from five databases were uploaded to Zotero, and duplicates were merged. After duplicates were removed, the remaining studies were uploaded to Rayyan, a software for systematic review, which was used by two independent reviewers (R.A.A. and S.A.) to screen titles and abstracts [28]. If needed, full texts were retrieved and reviewed to assess each study’s potential eligibility. If a conflict arose, the reviewers discussed and resolved any disagreements by consensus. The reviewer (R.A.A.) reviewed the full-text studies eligible for inclusion to ensure they met the predefined inclusion criteria.

### 2.7. Data Extraction

The first author (R.A.A.) extracted the data using a standardized data extraction form among all included studies. The data extraction form includes the following: author(s), publication year, country, study design, study setting, data source, study period, total sample size, sample size for older adults (≥65 years) with T2DM, participants’ ages, percentage of males, percentage of females, captured comorbidities, polypharmacy definition, polypharmacy prevalence, PIM criteria used and its version or year, PIM prevalence, PIM burden, top three most reported PIM classes, antidiabetic medication-related PIMs, and the proportion of patients with PIMs among those with polypharmacy. Some simple parameters (e.g., male and female percentages) were not reported in the included studies; the reviewer (R.A.A.) computed them based on the relevant parameters reported in the original research. No imputations were performed for missing data. Calculations assumed the reported denominator corresponded to the analyzed T2DM sample; if the denominator was unclear, the variable was not computed. For methodological rigor, another reviewer (A.A.A.) reviewed the extracted data, and both reviewers (R.A.A. and A.A.A.) discussed and resolved any disagreements by consensus.

### 2.8. Risk of Bias Assessment

Risk of bias of the included studies was assessed using the Joanna Briggs Institute (JBI) critical appraisal checklist for prevalence studies, which evaluates key domains relevant to prevalence research, including sampling methods, sample representativeness, measurement validity, and appropriateness of statistical analysis [29].

To enhance methodological rigor, overall risk-of-bias judgments were categorized as low, moderate, or high based on predefined decision criteria that considered both the total number of affirmative (“Yes”) responses and the presence of critical methodological flaws. Studies were classified as high risk of bias if they received at least one critical “No” rating in key domains (items 1, 2, 3, 6, or 7) or if they achieved four or fewer “Yes” responses overall, indicating substantial methodological limitations. Studies with eight to nine “Yes” responses and no critical “No” ratings were classified as low risk of bias, reflecting strong methodological robustness. All remaining studies, typically those with five to seven “Yes” responses or one to two “Unclear” ratings without critical flaws, were categorized as having a moderate risk of bias.

Each study was independently appraised by two reviewers (R.A.A. and A.A.A.). Discrepancies between reviewers’ judgments were resolved through discussion and consensus.

### 2.9. Data Synthesis and Statistical Analysis

A mixed-methods synthesis approach was employed. When studies were deemed sufficiently comparable in terms of population and outcome definitions, a random-effects meta-analysis was conducted. Pooled prevalence estimates of polypharmacy and PIM use among older adults with T2DM, along with corresponding 95% confidence intervals (CIs), were calculated across the included studies. For consistency across studies, polypharmacy was defined as the concurrent use of five or more medications. When a study reported medication burden in separate categories, such as polypharmacy (5–9 medications) and hyper-polypharmacy (≥10 medications), these categories were combined to derive a prevalence estimate consistent with the review definition for meta-analysis. Forest plots were generated to present study-specific prevalence estimates and the random-effects pooled estimate for each outcome. Meta-analyses were performed using R software, utilizing the meta package (version 8.3-0), the metadat package for meta-analysis datasets (version 1.4-0), and the metafor package (version 4.8-0).

Heterogeneity was assessed using the I^2^ statistic. I^2^ values were interpreted a priori using commonly applied in the meta-analysis literature (≤25% low, 26–50% moderate, 51–75% substantial, and >75% considerable heterogeneity) [30]. These thresholds were used as rough descriptive guides rather than absolute decision rules. When heterogeneity was extremely high, pooled prevalence estimates were interpreted with substantial caution as broad descriptive summaries rather than precise clinical benchmarks. Given the limited number of included studies per outcome, we did not conduct subgroup, sensitivity, or meta-regression analyses, nor did we assess the certainty of the evidence.

PIM burden and patterns were summarized using descriptive frequencies and proportions. When meta-analysis was not feasible due to insufficient data, findings were synthesized narratively and presented descriptively, consistent with the JBI manual for evidence synthesis [31].

## 3. Results

### 3.1. Study Selection

The systematic search identified 203 records across the included electronic databases. After removing duplicates, 91 records were screened for eligibility based on title and abstract, and 81 were excluded. Ten full-text records were retrieved and assessed for eligibility. Of these, five observational studies met the inclusion criteria and were included in the final synthesis [32,33,34,35,36]. Of the full-text reports, the remaining five studies were excluded: four for the absence of stratified data for older adults with T2DM [37,38,39,40], and one for not reporting the prevalence of polypharmacy and PIM use among the study participants [41].

**Figure 1:**
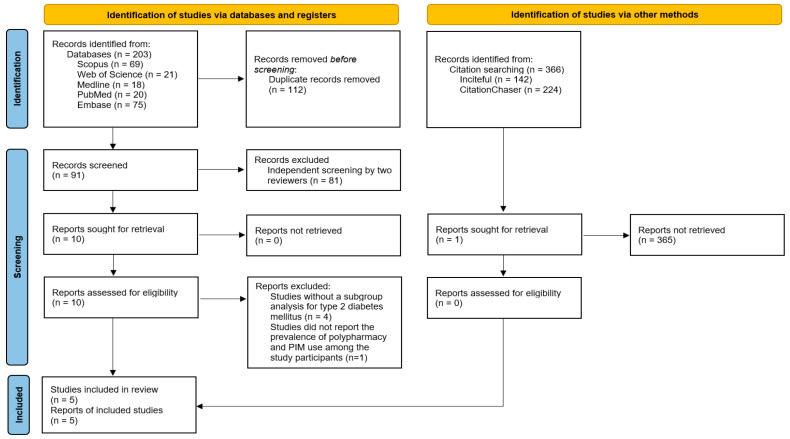
PRISMA 2020 flow diagram of the selection process.

In addition, citation searching using Inciteful and CitationChaser identified 366 records. After title and abstract screening, one record was identified and retrieved, but excluded as a duplicate of an already included record [34]. The study selection process is summarized in the PRISMA 2020 flow diagram (Figure 1).

### 3.2. Characteristics of Included Studies

A total of five observational studies with 13,350 older adults (≥65 years) with T2DM were included in this systematic review and meta-analysis [32,33,34,35,36]. They were published between 2020 and 2025 in four countries: the United Kingdom, Portugal (two studies), Iraq, and India. Four were cross-sectional studies [32,34,35,36], and one was a retrospective cohort study [33]. Study settings ranged from nationwide primary care or pharmacy databases to single tertiary hospitals and specialist diabetes and endocrine centers, with varying numbers of participants across studies. All studies included older adults with T2DM, defined as individuals aged 65 years and older. One study included middle-aged participants aged 45–64; however, a separate analysis was conducted for older adults aged 65 years and older [33].

Across the included studies, hypertension was the most commonly reported comorbidity and was identified in all five studies [32,33,34,35,36]. Dyslipidemia, cardiovascular diseases, and kidney-related disorders were also frequently reported across the included cohorts [32,33,34,35,36]. Common cardiovascular comorbidities included coronary heart disease, heart failure, myocardial infarction, coronary artery disease, acute coronary syndrome, and cerebrovascular disease [33,35,36]. Kidney-related conditions included chronic kidney disease, renal failure, and other renal disorders [33,34,35,36]. Respiratory diseases, psychiatric and neurological disorders, endocrine and metabolic conditions, gastrointestinal and liver diseases, and musculoskeletal disorders were also reported, although with greater variability across studies [32,33,34,35,36]. A more detailed, study-specific description is provided in Table 1.

### 3.3. Risk of Bias

According to the JBI critical appraisal criteria, three studies were rated as low risk of bias [33,34,35]. The other two studies were rated as high risk of bias [32,36] because they received critical “No” ratings on key JBI prevalence checklist items (Items 1 and 3: sample frame appropriateness and sample size adequacy). The risk-of-bias rate is presented in Table 1. In addition, a detailed risk-of-bias assessment table is available in the Supplementary Material File S3.

### 3.4. Definitions and Prevalence of Polypharmacy

All five studies reported the prevalence of polypharmacy among older adults with T2DM. The prevalence estimates ranged from 43.6% to 95.3%, with variation across healthcare settings, population characteristics, data sources, and prescribing practices. Each study estimate is summarized in Table 1.

Four studies explicitly defined polypharmacy as the use of five or more medications [32,33,34,35]. In one study, the operational threshold for polypharmacy was not clearly specified in the Methods section; however, medication burden was reported in two categories: (5–9 medications) and (≥10 medications) [36]. To ensure consistency with the review definition of polypharmacy and with the other studies included in the pooled prevalence analysis, these categories were combined to derive an overall estimate of polypharmacy prevalence. None of the included studies reported 95% CIs for prevalence estimates; therefore, study-level findings are presented as point estimates only.

**Figure 2:**
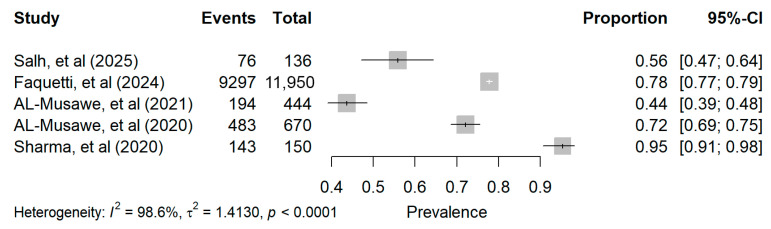
Forest plot of polypharmacy prevalence across the included studies. Squares represent study-specific polypharmacy prevalence estimates, and horizontal lines represent the corresponding 95% CIs. Note: The pooled estimate was not displayed due to very high between-study heterogeneity [32,33,34,35,36].

A random-effects meta-analysis was calculated; the pooled prevalence of polypharmacy among older adults with T2DM was 73% (95% CI 37–93%). This estimate should be interpreted with extreme caution because between-study heterogeneity was very high (I^2^ = 98.6%; 95% CI 98.0–99.1%) (Figure 2). Accordingly, this pooled estimate is presented as a broad descriptive summary only and should not be used as a precise prevalence estimate or clinical benchmark. Due to the limited number of included studies, exploration of the sources of heterogeneity was constrained.

### 3.5. PIMs

The detailed data in Table 1 summarize, across the five included studies, the study-level prevalence of PIMs, the assessment criteria used, including the version or year, PIM burden, the top three most reported PIM classes, antidiabetic medication-related PIMs, and the proportion of patients with PIMs among those with polypharmacy.

#### 3.5.1. Prevalence of PIMs and Assessment Criteria

The prevalence of PIMs among older adults with T2DM was reported in all included studies and varied widely across clinical settings and assessment criteria. Explicit validated criteria were used to identify PIMs, namely the AGS Beer’s criteria (2015 or 2019) and the STOPP criteria (version 2 or version 3). Two studies used the AGS Beers Criteria: one study applied the 2015 version [33], and the other applied the 2019 version [36]. On the other hand, three studies used the STOPP criteria (versions 2 or 3) [32,34,35]. Two applied version 2 [34,35], whereas one applied version 3 [32].

Across the included studies, the prevalence of PIMs ranged from 23.4% to 74% among older adults with T2DM (Table 1). None of the included studies reported the 95% CI for the prevalence of PIM use; therefore, study-level results are presented as point prevalence estimates only.

**Figure 3:**
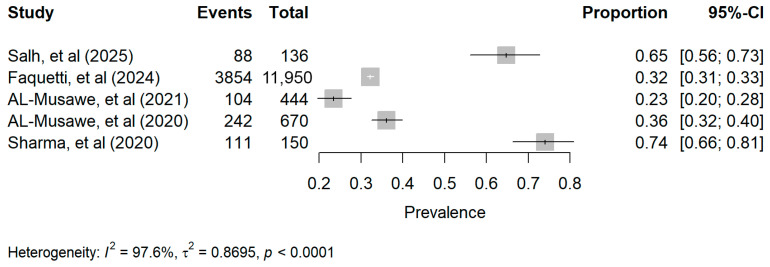
Forest plot of PIM prevalence across the included studies. Squares represent study-specific PIM prevalence estimates, and horizontal lines represent the corresponding 95% CIs. Note: The pooled estimate was not displayed due to very high between-study heterogeneity [32,33,34,35,36].

Using the random-effects model, the pooled prevalence of PIM use was 45% (95% CI 21–73%). However, this estimate should be interpreted with extreme caution because between-study heterogeneity was very high (I^2^ = 97.6%; 95% CI 96.2–98.5%) (Figure 3). Accordingly, this pooled estimate is presented as a broad descriptive summary only and should not be used as a precise prevalence estimate or clinical benchmark. Due to the limited number of included studies, exploration of potential sources of heterogeneity was constrained.

#### 3.5.2. PIM Burden

In addition to prevalence estimates, the burden of PIM use among older adults with T2DM was variably reported across studies. Direct comparisons of dose–response patterns were limited by inconsistent reporting categories for PIM counts (e.g., different thresholds such as ≥2 vs. ≥3 PIMs). Overall, most patients with PIM exposure were prescribed one PIM, although a substantial minority were exposed to multiple PIMs.

In the UK cohort, 22% of participants had one PIM, 6.1% had two PIMs, and approximately 4% had three or more PIMs [33]. In a Portuguese diabetes association cohort, 76.9% of patients had one PIM, 19.3% had two PIMs, and 3.8% had more than two PIMs [34]. Similarly, a Portuguese nationwide pharmacy cohort reported that 72.7% of patients had one PIM, 20.2% had two PIMs, and 7% had three or more PIMs [35].

In contrast, the Iraqi study found that 48.5% of patients had one PIM, and approximately 16% were exposed to two or more PIMs [32]. The Indian study reported that 38.7% of patients were prescribed one PIM, 24.7% were prescribed two PIMs, and 10.7% were prescribed three or more PIMs [36].

#### 3.5.3. Patterns of Common PIM Classes, Including Antidiabetic Medication-Related PIMs

Across the five included studies, several medication classes were consistently identified as potentially inappropriate for older adults with T2DM according to the AGS Beers Criteria or the STOPP criteria. The most commonly reported PIM class across the included studies was long-acting sulfonylureas, which were also the most frequently identified antidiabetic medication-related PIMs [32,33,34,35,36]. Proton pump inhibitors (PPIs) were also commonly identified, particularly when used for longer than 8 weeks [32,33,36]. Benzodiazepines were another recurrent PIM class reported across several studies [33,34,35]. In addition, angiotensin-converting enzyme inhibitors or angiotensin receptor blockers were prominently reported in some cohorts, particularly among patients with hyperkalemia [32,34].

Other reported PIMs included anticholinergic antidepressants and antipsychotics [33], human insulin prescribed solely according to random blood glucose measurements [36], and higher-dose iron supplements [35]. A more detailed study-specific analysis description of the reported PIM classes, including specific drug names where available, is provided in Table 1.

### 3.6. Narrative Summary of the Co-Occurrence of PIMs Among Those with Polypharmacy

All included studies reported the proportion of older adults with T2DM who had one or more PIMs among those classified as having polypharmacy (Table 1). Accordingly, consistent with guidance from the JBI Manual for evidence synthesis [31], the co-occurrence of PIMs among older adults with polypharmacy was summarized narratively, and no quantitative synthesis of association was performed.

Across studies, the proportion of patients with at least one PIM among those with polypharmacy ranged from 39.6% to 74% [32,33,34,35,36]. Specifically, this proportion was 39.6% in the UK primary care cohort [33], 45.3% in the 2020 Portuguese cohort [35], 73.7% in the Iraqi cohort [32], and 74% in the 2021 Portuguese diabetes association cohort [34]. In the Indian study, this proportion was reported separately: 36.7% among patients with 5–9 medications and 35.3% among those with more than 10 medications [36].

The Indian study was the only study to report comparative effect estimates, reporting an odds ratio (OR) of 2.82 (95% CI 0.58–13.52) and 7.85 (95% CI 1.49–41.10) for PIM exposure among older adults with T2DM in the 5–9 medications and more than 10 medications categories, respectively [36].

## 4. Discussion

This systematic review and meta-analysis synthesizes evidence on the prevalence of polypharmacy and PIM use among older adults (≥65 years) with T2DM. Beyond estimating prevalence, it integrates both outcomes within the same cohorts to clarify the co-occurrence of PIMs among polypharmacy patients and to describe the burden and patterns of PIM use in this population. Across the included studies, polypharmacy prevalence ranged from 43.6% to 95.3%, while PIM prevalence ranged from 23.4% to 74%, highlighting a significant medication burden among older adults with T2DM. Heterogeneity was extremely high for both polypharmacy (I^2^ = 98.6%, 95% CI 98–99.1%) and PIM use (I^2^ = 97.6%, 95% CI 96.2–98.5%). The included studies spanned diverse countries and clinical settings, used different data sources, and applied varying explicit criteria and versions to identify PIMs, indicating substantial between-study variability that likely contributed to the high heterogeneity. Therefore, the findings are best interpreted in terms of the wide range and overall pattern of prevalence reported across studies.

Direct comparisons of polypharmacy prevalence across studies remain limited by differences in thresholds, multimorbidity burden, and fragmentation of care [12,14]. However, the overall pattern of findings is consistent with previous evidence showing that polypharmacy is highly prevalent among older adults with diabetes [17,18]. Similarly, PIM prevalence estimates are constrained by heterogeneity in assessment criteria, which limits direct comparisons between studies [42]. The overall finding that PIM use is common among older adults with diabetes aligns with previous diabetes-specific studies. For example, Gagnon et al. found that 56% of older adults with diabetes used at least one PIM [43]. Likewise, Lu et al. reported that the prevalence of PIM among older adults with diabetes in China was 43.2%, 44.88%, and 42.40% in 2015, 2016, and 2017, respectively [44]. Compared with broader geriatric populations, older adults with diabetes appear to have a higher combined burden of polypharmacy and PIM exposure [45,46].

Our review found that hypertension was the most prevalent comorbidity among older adults with T2DM, followed by dyslipidemia, cardiovascular diseases, and chronic kidney disease. These results are consistent with prior evidence showing a high burden of cardiometabolic multimorbidity in this population [2,3]. Notably, these cardiometabolic conditions often cluster as metabolic syndrome, contributing to multimorbidity and a higher medication burden in older adults with T2DM [4,5,6]. Clinically, these findings are plausible and help explain the high prevalence of polypharmacy and PIM observed in this review, reflecting the complexity of T2DM management. Older adults with T2DM often require multidrug regimens for glycemic control and comorbidity management, as well as for guideline-recommended management of cardio-renal and vascular risk factors [11,47]. In addition, age-related pharmacokinetic and pharmacodynamic vulnerabilities may increase susceptibility to adverse drug reactions and drug–drug interactions, thereby progressively intensifying medication burden among older adults with T2DM [7,8].

These findings also suggest that redefining polypharmacy in T2DM care is necessary because several evolving factors highlight the limitations of traditional numerical definitions [12,14]. In T2DM care, medication counts alone are insufficient; polypharmacy must be interpreted in the clinical context, considering indications, benefit–risk balance, and treatment goals. Accordingly, fixed numerical thresholds (e.g., 5 or more medications) may not adequately distinguish between appropriate and inappropriate polypharmacy in T2DM patients [15,23]. Because management of multiple cardiovascular risk factors and comorbidities is central to preventing complications, an older adult receiving evidence-based therapies may readily meet numerical criteria for polypharmacy; in such cases, polypharmacy can be appropriate and guideline-concordant [11,15,47]. Conversely, the absence of polypharmacy may sometimes reflect under-prescribing and missed opportunities for comprehensive risk reduction and multimorbidity prevention [15,23,48]. Therefore, in T2DM care, the medication-count threshold used to flag polypharmacy may need to be interpreted flexibly, with an emphasis on identifying inappropriate polypharmacy rather than simply counting medicines. This is particularly relevant in older adults with T2DM, in whom treatment goals, frailty, comorbidity burden, and risk of hypoglycemia often necessitate individualized regimens and periodic reassessment of medication intensity [11,15,47]. In practice, structured medication review, supported by clinical pharmacy services, can help distinguish appropriate, evidence-based regimens from inappropriate polypharmacy by identifying PIMs, therapeutic duplication, and clinically significant drug–drug interaction risks [49,50].

Across the included studies, most older adults with T2DM who were prescribed PIMs were exposed to a single PIM; however, notable minorities received two or more PIMs, indicating a higher cumulative burden of potentially inappropriate prescribing and greater treatment complexity in this population [5,38]. Among the PIM classes identified, long-acting sulfonylureas, PPIs, and benzodiazepines were most reported, consistent with previous findings in older adults with diabetes [38,43,44]. Beyond PIM exposure, increasing medication burden in older adults with T2DM is associated with a higher risk of medication-related harm, including clinically significant drug–drug interactions and adverse drug effects [13,14,51]. These harms may also initiate prescribing cascades, in which a medication-related harm is misinterpreted as a new medical condition and treated with an additional drug, further escalating regimen complexity [8,9].

Importantly, the repeated identification of long-acting sulfonylureas aligns with updated explicit PIM criteria, given the heightened susceptibility of older adults to sulfonylurea-associated hypoglycemia and related adverse outcomes [26,27]. In this context, sulfonylurea-related hypoglycemia is also relevant to medication safety, because severe hypoglycemia has been linked to QTc prolongation in people with T2DM [52,53].

The proportion of patients with polypharmacy who were also prescribed at least one PIM varied widely across the included studies from 39.6% to 74%.

This descriptive pattern is broadly comparable to that reported in Al-Musawe et al.’s systematic review, which found that polypharmacy was associated with greater PIM exposure, with prevalence ranging from 22.7% to 79% using the Beers Criteria and from 48% to 79% using the STOPP criteria among older adults with T2DM [5]. Furthermore, broader evidence indicates that polypharmacy is an important correlate of PIM exposure among older adults [13,14]. In our review, only one study reported comparative effect estimates, and these were imprecise [36]. Notably, because most included studies did not report PIM prevalence among participants without polypharmacy within the same cohort, the true association between polypharmacy and PIM use could not be measured in this review. The limited availability of comparable effect measures precluded robust quantitative synthesis of the association and limited causal inference. This highlights an important evidence gap in T2DM care that should inform better treatment prioritization and targeted medication reviews.

Together, these findings suggest that polypharmacy and PIM exposure in older adults with T2DM represent important markers of medication burden and potentially modifiable targets for structured medication review and optimization. Explicit screening criteria, such as the Beers Criteria and STOPP/START criteria, can assist in identifying PIMs; however, their use should be integrated with individualized clinical judgment and patient-centered decision-making [54,55]. Importantly, the key evidence gap highlighted by this review is methodological rather than conceptual: differences in polypharmacy definitions, variation in PIM assessment criteria and their versions, and limitations in outcome reporting restrict direct comparisons across studies and constrain the interpretation and practical application of pooled prevalence estimates. Additionally, cross-country variation in diabetes treatment guidelines and their implementation, medication availability and formulary policies, and prescribing practices may further contribute to variability in polypharmacy patterns and PIM detection across studies. Accordingly, progress in this field requires standardized definitions and harmonized reporting of polypharmacy, PIM burden, and association measures, rather than relying solely on additional prevalence studies. In line with evidence-based guidelines for older adults with T2DM, medication optimization should remain patient-centered and may include deprescribing or treatment deintensification when potential harms outweigh benefits [51,56,57,58].

### 4.1. Strengths and Limitations

This systematic review has several methodological strengths. It provides a focused synthesis of evidence on polypharmacy and PIM use among older adults with T2DM. The review was conducted and reported in accordance with PRISMA 2020 guidelines and was prospectively registered with PROSPERO, thereby supporting transparency and minimizing the risk of selective reporting. The search was performed across five major medical databases and supplemented by automated citation searches. Additionally, the included studies estimated polypharmacy and PIM use within the same cohorts, which strengthened the assessment of their co-occurrence.

This review also has limitations. First, excluding non-English studies may have introduced language bias. Second, all included studies were observational, which limits causal inference regarding the association between polypharmacy and PIM use. Third, heterogeneity was high in pooled prevalence estimates for both polypharmacy and PIM use, likely reflecting differences across countries, settings, prescribing practices, populations, definitions of polypharmacy, and versions of PIM assessment criteria. Fourth, due to the small number of included studies and variability in methods, subgroup analyses, sensitivity analyses, and meta-regression were not performed; therefore, exploration of sources of heterogeneity and assessment of the robustness of pooled estimates were limited, despite completing a risk-of-bias assessment. Additionally, the association between polypharmacy and PIM use was summarized descriptively because only one study provided a comparative effect estimate, preventing pooled analysis of association measures. Lastly, medication exposure in most included studies was based on prescription and dispensing records; therefore, actual medication consumption and adherence remain uncertain. Moreover, formal validation of record accuracy and completeness was rarely reported, and variation in data sources and medication ascertainment periods across included studies limited standardization of medication exposure, which may have contributed to heterogeneity between studies.

### 4.2. Future Research

Future research should adopt standardized, diabetes-appropriate definitions of polypharmacy and clearly distinguish between appropriate and inappropriate polypharmacy. Observational studies should report clearly defined, adjusted effect estimates for PIM exposure among individuals with polypharmacy and use consistent reporting of PIM burden to enable assessment of potential dose–response patterns across studies. Despite searches across five major bibliographic databases, few eligible studies were identified, suggesting that evidence on polypharmacy and PIM use among older adults with T2DM is limited and underscoring the need for well-designed cohort studies in diverse settings. Finally, given the high burden of polypharmacy and PIMs identified in this review, intervention studies, particularly those evaluating pharmacist-led medication review, deprescribing, and diabetes treatment deintensification strategies tailored to older adults with T2DM, are needed to assess their effects on clinically meaningful outcomes such as hypoglycemia, falls, hospitalizations, and health-related quality of life.

## 5. Conclusions

Polypharmacy and PIM use are highly prevalent among older adults with T2DM, underscoring substantial treatment burden and clinical complexity. This medication burden also increases the potential for clinically meaningful drug–drug interactions and other medication-related adverse effects, which may contribute to prescribing cascades and further complicate treatment in this population. These findings highlight the need to redefine polypharmacy in T2DM care beyond a simple numerical threshold, as medication counts alone may misclassify guideline-concordant regimens and overlook clinically inappropriate prescribing. Accordingly, polypharmacy should be evaluated within the clinical context, including multimorbidity, frailty, renal function, individualized glycemic targets, and patient goals. Regular, structured medication reviews, informed by individualized clinical judgment and shared decision-making, are essential for identifying PIMs, optimizing regimens, and supporting deprescribing or treatment deintensification when potential harms outweigh benefits.

## Figures and Tables

**Table 1:** Summary of key characteristics across the five included studies.

Study (Authors, Year)	Country	Study Design	Study Setting	Data Source; Study Period	Total Sample Size	Sample Size (T2DM ≥65)	Participants’ Ages	Male (%)	Female (%)	Medications Received by Included Patients	Captured Comorbidities	Polypharmacy Definition	Polypharmacy Prevalence (%) ‡	PIM Prevalence (%) ‡	PIM Tool Used/ Version or Year	PIM Burden	Top Three Most Reported PIM Classes	Antidiabetic Medication-Related PIMs	Proportion of Patients with PIMs Among Those with Polypharmacy (%)	Risk of Bias
Salh et al. 2025 [32]	Iraq	Prospective, cross-sectional	Diabetes and endocrine disease center	Single center; 1 July to 1 September 2023	136	136	65 or more	34.6 *	65.4 *	Oral hypoglycemic agents, insulin, hypertensive agents, lipid-lowering agents, and antiplatelet agents, Proton Pump inhibitors (PPIs), other drugs; specific drug name was not reported	Hypertension, dyslipidemia, others	The use of 5 or more medicines	55.88	64.7	STOPP, V3	48.52% had one PIM, 13.97% had two PIMs, 1.47% had three PIMs, 0.75% had four PIMs	Long-acting sulfonylureas followed by PPIs for uncomplicated peptic ulcer disease at full therapeutic dosage for >8 weeks, then angiotensin-converting enzyme inhibitors or angiotensin receptor blockers in patients with hyperkalemia	Long-acting sulfonylureas; the specific drug name was not reported	73.7	High
Faquetti et al. 2024 [33]	United Kingdom	Retrospective, cohort	Primary care	Population-based primary care electronic medical database; 2016–2019	28,604	11,950	Middle-aged, 45–64, and older adults, aged 65 or more	55.7 *	44.2 *	Biguanides (metformin), sulfonylureas, dipeptidyl peptidase 4 inhibitors, sodium-glucose co-transporter inhibitors, thiazolidinediones, others; specific drug name was not reported	Hypertension, coronary heart disease, heart failure, cerebrovascular disease, chronic kidney disease, chronic liver disease, chronic obstructive pulmonary disease, asthma, sleep disorders, depression, alzheimer/dementia, hypothyroidism, osteoarthritis, osteoporosis	The prescription of ≥5 different drug compounds	77.8	32.25 *	AGS Beers, 2015	22% * had one PIM, 6.1%* had two PIMs, and approximately 4% * had three or more PIMs.	PPIs for >8 weeks followed by anticholinergic antidepressants as monotherapy or in combination, then antipsychotics, benzodiazepines, non-benzodiazepines, benzodiazepine receptor agonist hypnotics, tricyclic antidepressants, and selective serotonin reuptake inhibitors in patients with history of falls or fractures	Long-acting sulfonylureas (glyburide)	39.6	Low
AL-Musawe et al. 2021 [34]	Portugal	Cross-sectional	Database of the Portuguese diabetes association	Diabetes institution in Portugal	444	444	65 or more	55.85 *	44.14 *	Insulin, metformin, sulfonylureas, glucagon-like peptide-1 receptor agonist, dipeptidyl peptidase-4 inhibitor, sodium-glucose cotransporter-2 inhibitors; specific drug name was not reported	Hypertension, chronic kidney disease, dyslipidemia, and infections	The use of 5 or more medicines	43.6	23.4	STOPP, V2	76.9% had one PIM, 19.27% had two PIMs, 3.8% had more than two PIMs	Angiotensin-converting enzyme inhibitors or angiotensin receptor blockers in patients with hyperkalemia, followed by benzodiazepines, then long-acting sulfonylureas	Long-acting sulfonylureas; the specific drug name was not reported	74	Low
AL-Musawe et al. 2020 [35]	Portugal	Prospective, cross-sectional	All healthcare settings	Nationwide pharmacy-based cohort	1328	670	65 or more	50.45	49.55	Gliptins, insulin, glucagon-like peptide 1 receptor agonists, or sodium- glucose transport protein 2, renin- angiotensin system medicines, beta-blocking agents, diuretics, calcium channel blockers, lipid-lowering medicines, anti-thrombotic, acid-related disorders medicines, psycholeptics, psychoanaleptics; specific drug name was not reported	Hypertension, renal failure, heart failure, dyslipidemia, thyroid gland, respiratory system, digestive system, musculoskeletal system, prostate hyperplasia, neoplasms, depression, hyperuricemia, other	The use of 5 or more medicines	72.09	36.11	STOPP, V2	72.72% had one PIM, 20.24% had two PIMs, and 7.02% had more than two PIMs	Benzodiazepines followed by long-acting sulfonylureas (glibenclamide or glimepiride), then higher dose of iron supplements	Long-acting sulfonylureas (glibenclamide or glimepiride)	45.34	Low
Sharma et al. 2020 [36]	India	Prospective, cross-sectional	Tertiary hospital	Single center (hospitalized patients); August 2019 to January 2020	150	150	65 or more	54.7	45.3	Not reported	Hypertension, cerebrovascular accident, kidney disease, acute febrile illness, liver disease, myocardial infarction, dilated cardiomyopathy, coronary artery disease, left ventricular dysfunction, congestive heart failure, acute coronary syndrome, anemia, respiratory disease, psychiatric disorder, upper gastrointestinal bleeding, meningitis, urinary tract infection, hepatorenal syndrome, metabolic encephalopathy, benign prostate hyperplasia, obstructive uropathy, vertigo, others	The operational definitions of polypharmacy and hyper-polypharmacy were not explicitly stated in the study’s Methods section. The number of medications mentioned in the study’s Results section was grouped into two categories: 5–9 medications and ≥10 medications	54 (5–9 medications) category 41.3 (≥10 medications) category § The overall prevalence of polypharmacy 95.33	74	AGS Beers, 2019	38.7% had one PIM, 24.7% had two PIMs, and 10.7% had three or more PIMs	PPIs (omeprazole, pantoprazole, rabeprazole) followed by human insulin according to random blood sugar, then long-acting sulfonylureas (glimepiride)	Human insulin according to random blood sugar, followed by long-acting sulfonylureas (glimepiride)	36.7 (5–9 medications) category 35.3 (≥10 medications) category Only this study reports the association between polypharmacy and PIMs as an odds ratio (OR): OR 2.82 (95% CI 0.58–13.52). For (5–9 medications) category OR 7.85 (95% CI 1.49–41.10) For (≥10 medications) category	High

* Items were computed by the reviewer (R.A.A) based on data presented in the study tables of the included studies. No imputations were performed for missing data. ‡ Confidence intervals were not reported in the original studies and were therefore not presented. § In this study, the prevalence of polypharmacy (5–9 medications) and hyper-polypharmacy (≥10 medications) was reported separately. To ensure consistency with the other studies included in the pooled prevalence analysis, these categories were combined to derive an overall estimate of polypharmacy prevalence. PIM: potentially inappropriate medication. OR: odds ratio.

## Data Availability

No new data were created or analyzed in this study.

## Supplementary Materials

The following supporting information can be downloaded at: https://www.mdpi.com/article/10.3390/pharmacy14030065/s1, Supplementary File S1: The PRISMA 2020 checklist for this review; Supplementary File S2: The search syntax used for this review; Supplementary File S3: A detailed risk of bias assessment table.

## Abbreviations

The following abbreviations are used in this manuscript:

AGS	American Geriatrics Society
CI	Confidence Interval
JBI	Joanna Briggs Institute
OR	Odds Ratio
PIM	Potential Inappropriate Medication
PPIs	Proton Pump Inhibitors
STOPP/ START	Screening Tool of Older Persons’ Potentially Prescriptions/Screening Tool to Alert to Right Treatment criteria
T2DM	Type 2 Diabetes Mellitus
