# Supplementary material for: Prevalence and Association of Polypharmacy and Potentially Inappropriate Medications Among Older Adults with Type 2 Diabetes Mellitus: A Systematic Review and Meta-Analysis
Source: Pharmacy (Basel). 2026 Apr 28;14(3):65. doi: 10.3390/pharmacy14030065 (PMC13214745; doi:10.3390/pharmacy14030065)
Supplement: Supplementary file 1 [file pharmacy-14-00065-s001.zip › S2_Polypharmacy and PIM- The search syntax used for this review.pdf]

The search syntax used for this review

Scopus

TITLE-ABS-KEY ((polypharm* OR "multiple medication*" OR "multiple drug*" OR "multi-drug*" OR "concurrent use of multiple medicine*") AND ("potentially inappropriate medication*" OR "potentially inappropriate medicine*" OR "potentially inappropriate prescri*" OR "inappropriate prescri*" OR "inappropriate medication use" OR (PIM* W/3 (medicat* OR prescri*)) OR "medication appropriateness index" OR MAI OR Beers) AND ("type 2 diabet*" OR T2DM OR "type II diabet*" OR NIDDM OR "noninsulin-dependent diabet*" OR "non insulin dependent diabet*" OR "adult-onset diabet*")) AND (LIMIT-TO (DOCTYPE , "ar")) AND (LIMIT-TO (LANGUAGE , "English"))

Web of Science and Medline

TS= ((polypharm* OR "multiple medication*" OR "multiple drug*" OR "multi-drug*" OR "concurrent use of multiple medicine*") AND ("potentially inappropriate medication*" OR "potentially inappropriate medicine*" OR "potentially inappropriate prescri*" OR "inappropriate prescri*" OR "inappropriate medication use" OR (PIM* W/3 (medicat* OR prescri*)) OR "medication appropriateness index" OR MAI OR Beers) AND ("type 2 diabet*" OR T2DM OR "type II diabet*" OR NIDDM OR "noninsulin-dependent diabet*" OR "non insulin dependent diabet*" OR "adult-onset diabet*"))

PubMed

(("Polypharmacy"[MeSH Terms] OR polypharm*[tiab] OR "multiple medication*"[tiab] OR "multiple drug*"[tiab] OR "multi-drug*"[tiab] OR "concurrent use of multiple medicine*"[tiab]) AND ("Inappropriate Prescribing"[MeSH Terms] OR "Potentially Inappropriate Medication List"[MeSH Terms] OR "potentially inappropriate medication*"[tiab] OR "potentially inappropriate medicine*"[tiab] OR "potentially inappropriate prescri*"[tiab] OR "inappropriate prescri*"[tiab] OR "inappropriate medication use"[tiab] OR (PIM*[tiab] AND (medicat*[tiab] OR prescri*[tiab])) OR "medication appropriateness index"[tiab] OR MAI[tiab] OR Beers[tiab]) AND ("Diabetes Mellitus, Type 2"[MeSH Terms] OR "type 2 diabet*"[tiab] OR T2DM[tiab] OR "type II diabet*"[tiab] OR NIDDM[tiab] OR "noninsulin-dependent diabet*"[tiab] OR "non insulin dependent diabet*"[tiab] OR "adult-onset diabet*"[tiab]))

Embase

('polypharmacy'/exp OR polypharm*:ti,ab,kw OR 'multiple medication*':ti,ab,kw OR 'multiple drug*':ti,ab,kw OR 'multi-drug*':ti,ab,kw OR 'concurrent use of multiple medicine*':ti,ab,kw) AND ('inappropriate prescribing'/exp OR 'potentially inappropriate medication'/exp OR 'potentially inappropriate medication*':ti,ab,kw OR 'potentially inappropriate medicine*':ti,ab,kw OR 'potentially inappropriate prescri*':ti,ab,kw OR 'inappropriate prescri*':ti,ab,kw OR 'inappropriate medication use':ti,ab,kw OR PIM*:ti,ab,kw OR 'medication appropriateness index':ti,ab,kw OR MAI:ti,ab,kw OR Beers:ti,ab,kw) AND ('type 2 diabetes mellitus'/exp OR 'type 2 diabet*':ti,ab,kw OR T2DM:ti,ab,kw OR 'type II diabet*':ti,ab,kw OR NIDDM:ti,ab,kw OR 'noninsulin-dependent diabet*':ti,ab,kw OR 'non insulin dependent diabet*':ti,ab,kw OR 'adult-onset diabet*':ti,ab,kw)
